# 6-(3,5-Dimeth­oxy­benzyl­amino)-9-(oxan-2-yl)-9*H*-purine

**DOI:** 10.1107/S1600536813006697

**Published:** 2013-03-13

**Authors:** Pavel Štarha, Igor Popa, Zdeněk Dvořák, Zdeněk Trávníček

**Affiliations:** a Department of Inorganic Chemistry, Faculty of Science, Palacký University, 17. listopadu 12, CZ-771 46 Olomouc, Czech Republic; b Department of Cell Biology and Genetics, Faculty of Science, Palacký University, Šlechtitelů 11, CZ-783 71 Olomouc, Czech Republic

## Abstract

The mol­ecule of the title compound, C_19_H_23_N_5_O_3_, contains six-membered pyrimidine and five-membered imidazole rings merged into the essentially planar purine skeleton (r.m.s. deviation = 0.01 Å). In the crystal, pairs of N—H⋯N hydrogen bonds link mol­ecules into inversion dimers. The dimers are linked *via* C—H⋯O hydrogen bonds, forming double-stranded chains propagating along [001]. These chains are linked *via* C—H⋯π and parallel slipped π–π inter­actions [centroid–centroid distance = 3.467 (1) Å; slippage 0.519 Å], forming a three-dimensional network.

## Related literature

For the alternative synthetic procedure and biological activity of the title compound, see: Szüčová *et al.* (2009 ▶). For the structures of similar compounds, see: Soriano-Garcia *et al.* (2003 ▶); Taddei *et al.* (2004 ▶). For puckering parameters, see: Cremer & Pople (1975 ▶).
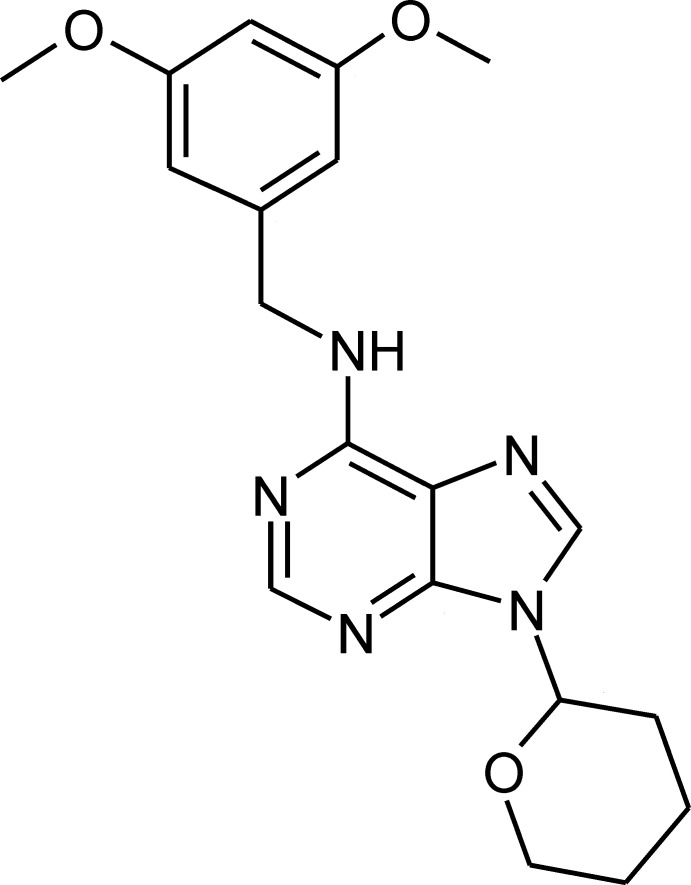


## Experimental

### 

#### Crystal data

C_19_H_23_N_5_O_3_
*M* *_r_* = 369.42
Triclinic, 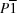

*a* = 8.6978 (3) Å
*b* = 8.8318 (3) Å
*c* = 12.1517 (4) Å
α = 84.808 (3)°
β = 78.674 (3)°
γ = 82.039 (3)°
*V* = 904.53 (5) Å^3^
*Z* = 2
Mo *K*α radiation
μ = 0.10 mm^−1^
*T* = 110 K
0.40 × 0.40 × 0.35 mm

#### Data collection

Agilent Xcalibur Sapphire2 diffractometer
Absorption correction: multi-scan (*CrysAlis PRO*; Agilent, 2012 ▶). *T*_min_ = 0.963, *T*_max_ = 0.968
6684 measured reflections
3177 independent reflections
2784 reflections with *I* > 2σ(*I*)
*R*_int_ = 0.010

#### Refinement

*R*[*F* ^2^ > 2σ(*F* ^2^)] = 0.044
*wR*(*F* ^2^) = 0.116
*S* = 1.07
3177 reflections
246 parameters
H-atom parameters constrained
Δρ_max_ = 0.45 e Å^−3^
Δρ_min_ = −0.22 e Å^−3^

### 

Data collection: *CrysAlis PRO* (Agilent, 2012 ▶); cell refinement: *CrysAlis PRO*; data reduction: *CrysAlis PRO*; program(s) used to solve structure: *SHELXS97* (Sheldrick, 2008 ▶); program(s) used to refine structure: *SHELXL97* (Sheldrick, 2008 ▶); molecular graphics: *DIAMOND* (Brandenburg, 2011 ▶); software used to prepare material for publication: *publCIF* (Westrip, 2010 ▶).

## Supplementary Material

Crystal structure: contains datablock(s) I, global. DOI: 10.1107/S1600536813006697/zq2196sup1.cif

Structure factors: contains datablock(s) I. DOI: 10.1107/S1600536813006697/zq2196Isup2.hkl

Supplementary material file. DOI: 10.1107/S1600536813006697/zq2196Isup3.cml

Additional supplementary materials:  crystallographic information; 3D view; checkCIF report

## Figures and Tables

**Table 1:** Hydrogen-bond geometry (Å, °) *Cg* is the centroid of the C10–C15 ring.

*D*—H⋯*A*	*D*—H	H⋯*A*	*D*⋯*A*	*D*—H⋯*A*
N6—H6⋯N7^i^	0.88	2.38	3.155 (2)	147
C17—H17*A*⋯O3^ii^	0.98	2.44	3.216 (3)	136
C8—H8⋯*Cg* ^i^	0.95	2.72	3.506 (2)	142
C21—H21*B*⋯*Cg* ^iii^	0.99	2.93	3.904 (3)	169

Symmetry codes: (i) 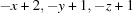; (ii) 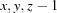; (iii) 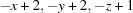.

**Fig. 1.**
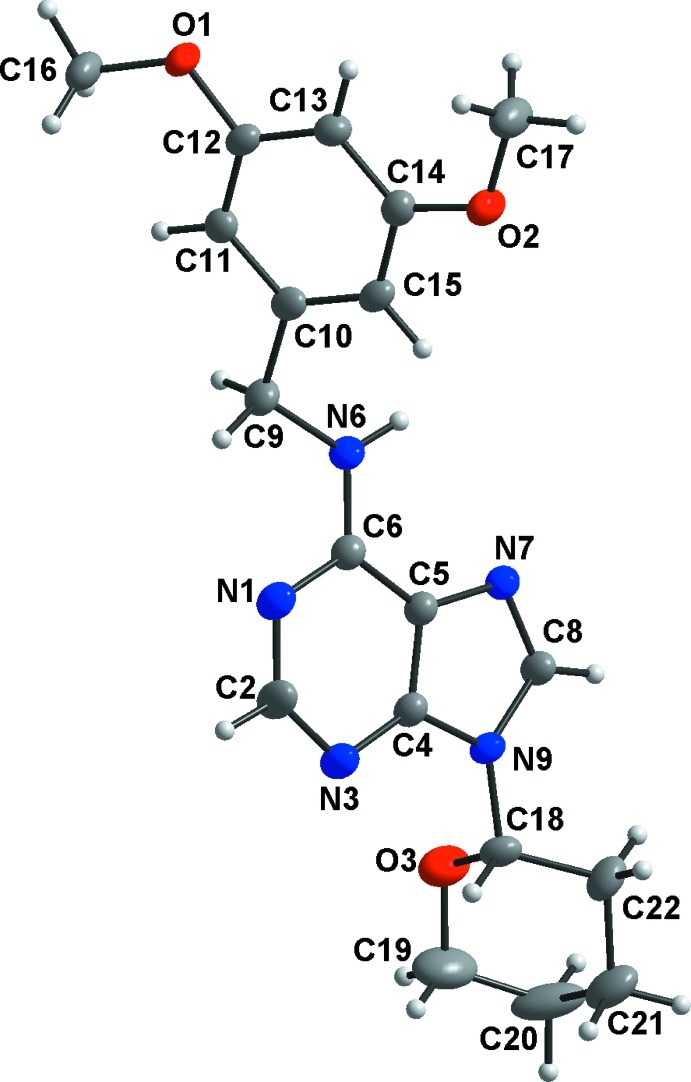
The molecular structure of the title compound with the non-hydrogen atoms depicted as thermal ellipsoids at the 50% probability level and given with the atom numbering scheme.

**Fig. 2.**
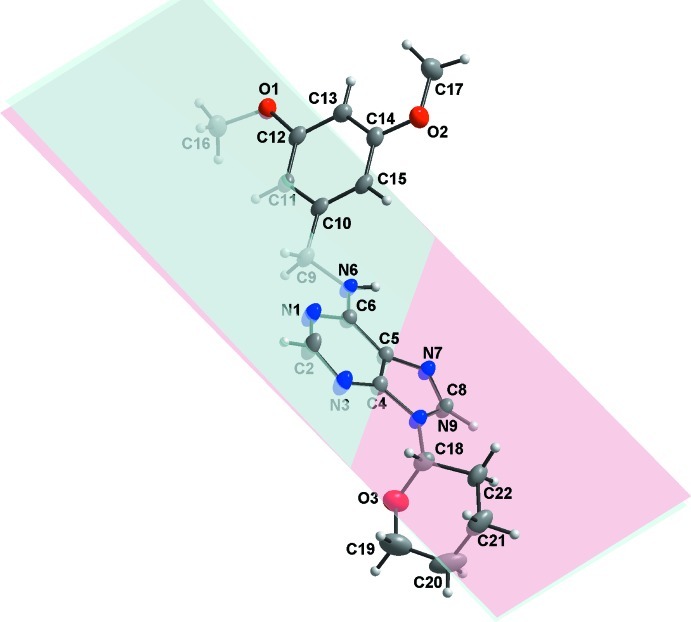
The model of the title compound, showing the mutual orientation of the six-membered pyrimidine (least-squares plane created through the N1, C2, N3, C4, C5 and C6 atoms; in blue) and five-membered imidazole rings (least-squares plane created through the C4, C5, N7, C8 and N9 atoms; in red). The planes are nearly coplanar forming the dihedral angle of 1.50 (6)°.

**Fig. 3.**
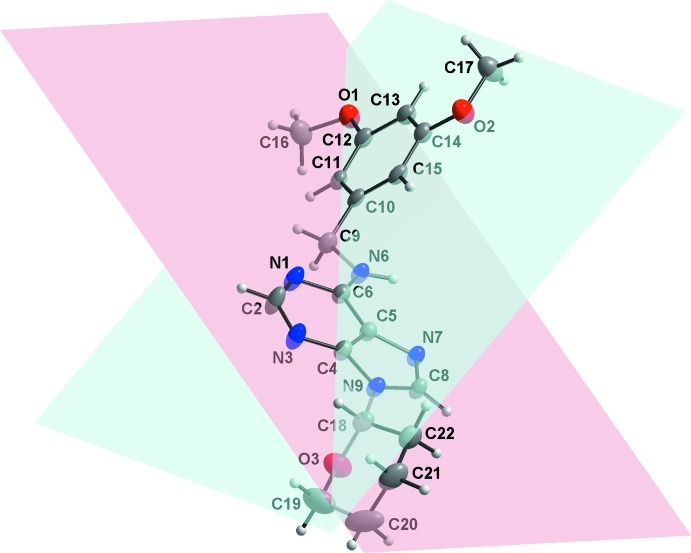
The model of the title compound, showing the mutual orientation of the purine skeleton (least-squares plane created through the N1, C2, N3, C4, C5, C6, N7, C8 and N9 atoms; in red) and benzene ring (least-squares plane created through the C10, C11, C12, C13, C14 and C15 atoms; in blue). The planes form the dihedral angle of 63.87 (4)°.

**Fig. 4.**
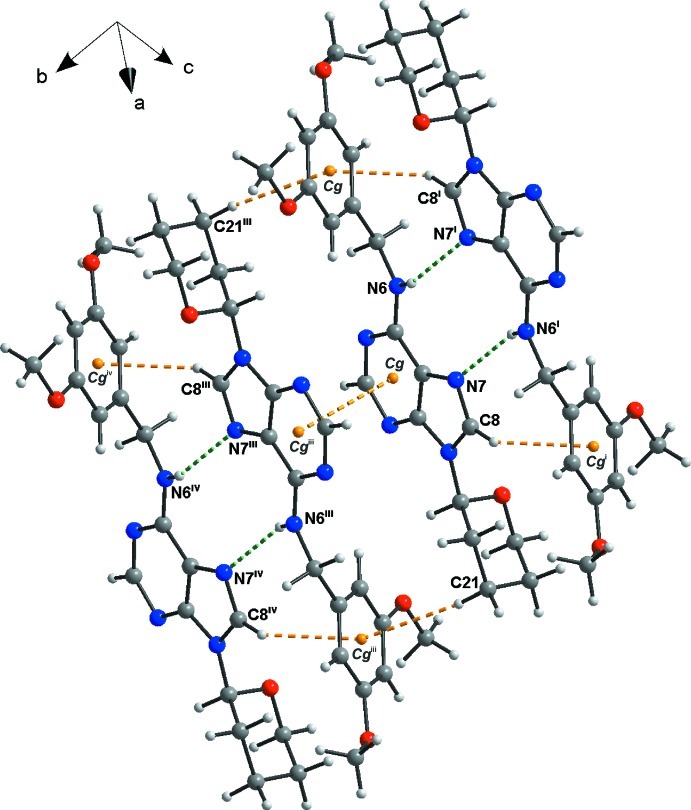
Part of the crystal structure of the title compound (Ball-and-stick model), showing the N6—H6···N7 hydrogen bonds (dashed green lines; see Table 1 for parameters) and C8—H8···π, C21—H21···π and π···π interactions (dashed orange lines; see Table 1 for C8—H8···Cg and C21—H21···Cg parameters). Cg···Cgiii parameters: d(D···A) = 3.46700 (10) Å. Symmetry codes: (i) –x+2, –y+1, –z+1; (iii) –x+2, –y+2, –z+1; (iv) x, y+1, z.

**Fig. 5.**
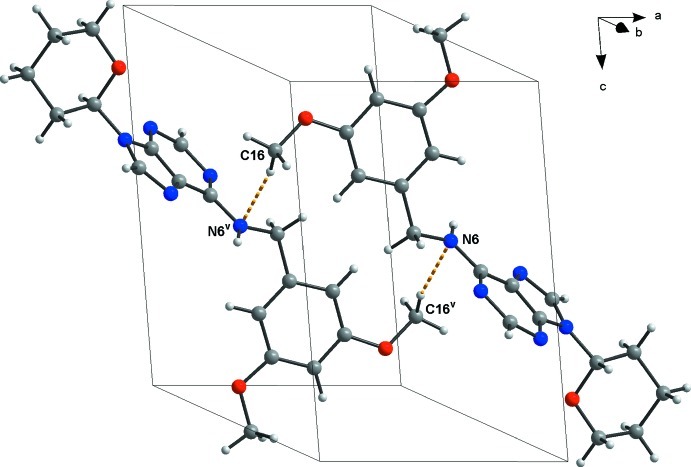
Part of the crystal structure of the title compound (Ball-and-stick model), showing two molecules connected through C16—H16C···N6 non-covalent contacts (dashed orange lines; see Table 1 for parameters). Symmetry code: (v) –x+1, –y+1, –z+1.

## supplementary crystallographic information

### Comment

The title compound, 6-(3,5-dimethoxybenzylamino)-9-(tetrahydropyran-2-yl)-9*H*-purine (Fig. 1), is formed by the essentially planar purine moiety substituted by 3,5-dimethoxybenzylamine and tetrahydropyran-2-yl at the C6, and N9 position, respectively (for related structures, see: Soriano-Garcia *et al.*, 2003; Taddei *et al.*, 2004). The six-membered pyrimidine and five-membered imidazole rings of the purine moiety form the dihedral angle of 1.50 (6)° (Fig. 2), while the planes fitted through all the non-hydrogen atoms of purine and benzene rings form the dihedral angle of 63.87 (4)° (Fig. 3). The most deviated atoms from the planes created through the pyrimidine, imidazole, purine and benzene rings are C5 (0.006 (2) Å), C8 (-0.005 (2) Å), C5 (0.021 (2) Å), and C12 (0.011 (2) Å), respectively. The crystal structure is stabilized by the N6—H6···N7 hydrogen bonds (see Table 1 for parameters) and C—H···O, C—H···π and parallel slipped π–π interactions [centroid–centroid distance = 3.467 (1) Å; slippage 0.519 Å] (Fig. 4 and Fig. 5). The tetrahydropyranyl ring adopts a chair conformation. The Cremer-Pople puckering parameters (Cremer & Pople, 1975) are Q_T_ = 0.691 (2) Å, Θ_2_ = 89.4 (2)°, q_2_ = 0.691 (2)°, q_3_ = 0.007 (2)° and φ_2_ = 148.8 (2)°.

### Experimental

The title compound, synthesized with the aim of its possible utilization as a suitable ligand in coordination chemistry of transition metals, was prepared by a slightly modified method reported by Szüčová *et al.*, 2009. The starting compounds 6-chloropurine and 3,4-dihydro-2*H*-pyrane (a molar ratio of 1:2) were stirred in a minimum volume of ethanol (15 min, laboratory temperature) and then CF_3_COOH (1.30 molar equivalent of 6-chloropurine) was slowly poured. The reaction mixture was neutralized by 10% NH_4_OH after 24 h of stirring at laboratory temperature. The solvents were evaporated and yellowish product was washed (distilled water, methanol, diethyl ether) and dried in desiccator over P_4_O_10_. The obtained 6-chloro-9-(tetrahydropyran-2-yl)-9*H*-purine was dissolved in a minimum volume of *N,N`*-dimethylformamide and 3,5-dimethoxybenzylamine (1.33 molar equivalent) and triethylamine (1.67 molar equivalent) were poured in. The reaction mixture was stirred at 90 °C for 150 min and then it was evaporated to dryness. The solid was suspended in cold distilled water, filtered off, washed (distilled water, methanol, diethyl ether) and dried in desiccator over P_4_O_10_. The powder product was recrystallized from ethanol and the obtained microcrystals, suitable for single-crystal X-ray analysis, were collected by filtration. Analysis calculated for C_19_H_23_N_5_O_3_: C 61.8, H 6.3, N 19.0%; found: C 61.7, H 6.4, N 18.8%. Elemental analysis (C, H, N) was performed on a Thermo Scientific Flash 2000 CHNO-S Analyzer.

### Refinement

Non-hydrogen atoms were refined anisotropically and hydrogen atoms were located in difference maps and refined using the riding model with C—H = 0.95 (CH), C—H = 0.99 (CH_2_), C—H = 0.98 (CH_3_) Å, and N—H = 0.88 Å, with *U*_iso_(H) = 1.2*U*_eq_(CH, CH_2_, NH) and 1.5*U*_eq_(CH_3_). The maximum and minimum residual electron density peaks of 0.45 and -0.22 e Å^-3^, respectively, were located 0.93 Å and 0.74 Å from the C21 and C19 atoms, respectively.

### Crystal data

**Table:**

C_19_H_23_N_5_O_3_	*Z* = 2
*M**_r_* = 369.42	*F*(000) = 392
Triclinic, *P*1	*D*_x_ = 1.356 Mg m^−^^3^
Hall symbol: -P 1	Mo *K*α radiation, λ = 0.71073 Å
*a* = 8.6978 (3) Å	Cell parameters from 6498 reflections
*b* = 8.8318 (3) Å	θ = 3.0–31.7°
*c* = 12.1517 (4) Å	µ = 0.10 mm^−^^1^
α = 84.808 (3)°	*T* = 110 K
β = 78.674 (3)°	Prism, colourless
γ = 82.039 (3)°	0.40 × 0.40 × 0.35 mm
*V* = 904.53 (5) Å^3^	

### Data collection

**Table:**

Agilent Xcalibur Sapphire2 diffractometer	3177 independent reflections
Radiation source: Enhance (Mo) X-ray Source	2784 reflections with *I* > 2σ(*I*)
Graphite monochromator	*R*_int_ = 0.010
Detector resolution: 8.3611 pixels mm^-1^	θ_max_ = 25.0°, θ_min_ = 3.0°
ω scans	*h* = −8→10
Absorption correction: multi-scan (*CrysAlis PRO*; Agilent, 2012).	*k* = −10→9
*T*_min_ = 0.963, *T*_max_ = 0.968	*l* = −14→14
6684 measured reflections	

### Refinement

**Table:**

Refinement on *F*^2^	Primary atom site location: structure-invariant direct methods
Least-squares matrix: full	Secondary atom site location: difference Fourier map
*R*[*F*^2^ > 2σ(*F*^2^)] = 0.044	Hydrogen site location: inferred from neighbouring sites
*wR*(*F*^2^) = 0.116	H-atom parameters constrained
*S* = 1.07	*w* = 1/[σ^2^(*F*_o_^2^) + (0.0525*P*)^2^ + 0.5406*P*] where *P* = (*F*_o_^2^ + 2*F*_c_^2^)/3
3177 reflections	(Δ/σ)_max_ < 0.001
246 parameters	Δρ_max_ = 0.45 e Å^−^^3^
0 restraints	Δρ_min_ = −0.22 e Å^−^^3^

### Special details

**Table:**

Geometry. All e.s.d.'s (except the e.s.d. in the dihedral angle between two l.s. planes) are estimated using the full covariance matrix. The cell e.s.d.'s are taken into account individually in the estimation of e.s.d.'s in distances, angles and torsion angles; correlations between e.s.d.'s in cell parameters are only used when they are defined by crystal symmetry. An approximate (isotropic) treatment of cell e.s.d.'s is used for estimating e.s.d.'s involving l.s. planes.
Refinement. Refinement of *F*^2^ against ALL reflections. The weighted *R*-factor *wR* and goodness of fit *S* are based on *F*^2^, conventional *R*-factors *R* are based on *F*, with *F* set to zero for negative *F*^2^. The threshold expression of *F*^2^ > σ(*F*^2^) is used only for calculating *R*-factors(gt) *etc*. and is not relevant to the choice of reflections for refinement. *R*-factors based on *F*^2^ are statistically about twice as large as those based on *F*, and *R*- factors based on ALL data will be even larger.

### Fractional atomic coordinates and isotropic or equivalent isotropic displacement parameters (Å^2^)

**Table:**

	*x*	*y*	*z*	*U*_iso_*/*U*_eq_	
O1	0.34333 (13)	0.54945 (15)	0.18751 (10)	0.0325 (3)	
N1	0.74662 (17)	0.93176 (17)	0.56497 (14)	0.0343 (4)	
O2	0.86794 (14)	0.66871 (15)	0.06254 (11)	0.0348 (3)	
C2	0.7939 (2)	1.0254 (2)	0.62854 (19)	0.0393 (5)	
H2	0.7252	1.1178	0.6431	0.047*	
N3	0.92245 (18)	1.00924 (17)	0.67466 (14)	0.0347 (4)	
O3	1.14994 (17)	0.82537 (18)	0.87030 (12)	0.0457 (4)	
C4	1.01114 (19)	0.87587 (19)	0.64793 (14)	0.0253 (4)	
C5	0.98030 (19)	0.76782 (18)	0.58273 (14)	0.0240 (4)	
N6	0.79042 (16)	0.70162 (17)	0.47898 (12)	0.0289 (3)	
H6	0.8574	0.6235	0.4525	0.035*	
C6	0.83853 (19)	0.79912 (19)	0.54048 (14)	0.0260 (4)	
N7	1.09793 (16)	0.64320 (16)	0.57585 (12)	0.0269 (3)	
C8	1.19489 (19)	0.67977 (19)	0.63655 (15)	0.0265 (4)	
H8	1.2878	0.6148	0.6480	0.032*	
N9	1.15153 (16)	0.81862 (15)	0.68192 (12)	0.0250 (3)	
C9	0.6313 (2)	0.7216 (2)	0.45512 (15)	0.0302 (4)	
H9A	0.5858	0.8299	0.4635	0.036*	
H9B	0.5652	0.6577	0.5113	0.036*	
C10	0.62444 (19)	0.67918 (18)	0.33892 (14)	0.0245 (4)	
C11	0.48482 (19)	0.63085 (18)	0.32182 (14)	0.0244 (4)	
H11	0.3985	0.6226	0.3826	0.029*	
C12	0.47421 (19)	0.59515 (18)	0.21484 (15)	0.0251 (4)	
C13	0.6021 (2)	0.60276 (19)	0.12598 (15)	0.0270 (4)	
H13	0.5954	0.5746	0.0536	0.032*	
C14	0.73869 (19)	0.65179 (19)	0.14429 (15)	0.0262 (4)	
C15	0.75085 (19)	0.69009 (18)	0.25110 (14)	0.0255 (4)	
H15	0.8452	0.7233	0.2632	0.031*	
C16	0.2009 (2)	0.5642 (2)	0.26940 (16)	0.0374 (5)	
H16A	0.1155	0.5309	0.2391	0.056*	
H16B	0.1729	0.6716	0.2877	0.056*	
H16C	0.2164	0.5004	0.3375	0.056*	
C17	0.8598 (2)	0.6274 (3)	−0.04723 (16)	0.0421 (5)	
H17A	0.9599	0.6404	−0.0984	0.063*	
H17B	0.7734	0.6933	−0.0751	0.063*	
H17C	0.8403	0.5202	−0.0433	0.063*	
C18	1.2218 (2)	0.88515 (19)	0.76217 (15)	0.0278 (4)	
H18	1.1926	0.9988	0.7569	0.033*	
C19	1.2010 (3)	0.8915 (3)	0.95875 (19)	0.0599 (7)	
H19A	1.1479	0.8498	1.0327	0.072*	
H19B	1.1712	1.0039	0.9541	0.072*	
C20	1.3771 (3)	0.8561 (3)	0.9489 (2)	0.0600 (7)	
H20A	1.4064	0.7439	0.9578	0.072*	
H20B	1.4112	0.9037	1.0093	0.072*	
C21	1.4591 (3)	0.9167 (3)	0.8360 (2)	0.0486 (6)	
H21A	1.5746	0.8868	0.8279	0.058*	
H21B	1.4389	1.0300	0.8302	0.058*	
C22	1.3974 (2)	0.8509 (2)	0.74142 (18)	0.0381 (5)	
H22A	1.4431	0.8983	0.6674	0.046*	
H22B	1.4289	0.7388	0.7412	0.046*	

### Atomic displacement parameters (Å^2^)

**Table:**

	*U*^11^	*U*^22^	*U*^33^	*U*^12^	*U*^13^	*U*^23^
O1	0.0209 (6)	0.0443 (8)	0.0359 (7)	−0.0083 (5)	−0.0071 (5)	−0.0122 (6)
N1	0.0259 (8)	0.0266 (8)	0.0536 (10)	0.0034 (6)	−0.0173 (7)	−0.0084 (7)
O2	0.0270 (7)	0.0448 (8)	0.0353 (7)	−0.0124 (6)	−0.0040 (5)	−0.0088 (6)
C2	0.0283 (10)	0.0256 (9)	0.0678 (14)	0.0061 (7)	−0.0193 (9)	−0.0143 (9)
N3	0.0269 (8)	0.0245 (8)	0.0561 (10)	0.0023 (6)	−0.0157 (7)	−0.0123 (7)
O3	0.0487 (9)	0.0562 (9)	0.0373 (8)	−0.0194 (7)	−0.0102 (6)	−0.0057 (7)
C4	0.0207 (8)	0.0224 (8)	0.0341 (9)	−0.0019 (6)	−0.0082 (7)	−0.0033 (7)
C5	0.0209 (8)	0.0224 (8)	0.0295 (9)	−0.0012 (6)	−0.0070 (7)	−0.0029 (7)
N6	0.0231 (7)	0.0306 (8)	0.0360 (8)	0.0043 (6)	−0.0145 (6)	−0.0101 (6)
C6	0.0225 (8)	0.0257 (9)	0.0310 (9)	−0.0008 (7)	−0.0084 (7)	−0.0031 (7)
N7	0.0221 (7)	0.0256 (7)	0.0347 (8)	0.0019 (6)	−0.0109 (6)	−0.0068 (6)
C8	0.0227 (8)	0.0219 (8)	0.0367 (9)	0.0018 (6)	−0.0115 (7)	−0.0054 (7)
N9	0.0221 (7)	0.0213 (7)	0.0344 (8)	−0.0014 (5)	−0.0118 (6)	−0.0046 (6)
C9	0.0217 (8)	0.0357 (10)	0.0352 (10)	0.0016 (7)	−0.0116 (7)	−0.0069 (8)
C10	0.0233 (8)	0.0177 (8)	0.0340 (9)	0.0040 (6)	−0.0132 (7)	−0.0034 (7)
C11	0.0214 (8)	0.0205 (8)	0.0316 (9)	0.0022 (6)	−0.0078 (7)	−0.0039 (7)
C12	0.0215 (8)	0.0202 (8)	0.0363 (9)	−0.0013 (6)	−0.0119 (7)	−0.0044 (7)
C13	0.0264 (9)	0.0259 (9)	0.0312 (9)	−0.0017 (7)	−0.0104 (7)	−0.0059 (7)
C14	0.0224 (8)	0.0223 (8)	0.0341 (9)	−0.0014 (6)	−0.0064 (7)	−0.0026 (7)
C15	0.0214 (8)	0.0200 (8)	0.0378 (10)	−0.0006 (6)	−0.0131 (7)	−0.0027 (7)
C16	0.0238 (9)	0.0543 (12)	0.0373 (10)	−0.0112 (8)	−0.0048 (8)	−0.0116 (9)
C17	0.0347 (10)	0.0602 (13)	0.0348 (10)	−0.0167 (9)	−0.0037 (8)	−0.0096 (9)
C18	0.0311 (9)	0.0207 (8)	0.0360 (10)	−0.0055 (7)	−0.0144 (8)	−0.0031 (7)
C19	0.0812 (18)	0.0688 (16)	0.0379 (12)	−0.0238 (14)	−0.0180 (12)	−0.0101 (11)
C20	0.0923 (19)	0.0450 (13)	0.0620 (15)	−0.0279 (13)	−0.0523 (14)	0.0076 (11)
C21	0.0400 (12)	0.0424 (12)	0.0728 (16)	−0.0082 (9)	−0.0319 (11)	−0.0014 (11)
C22	0.0238 (9)	0.0453 (11)	0.0477 (12)	−0.0038 (8)	−0.0140 (8)	−0.0015 (9)

### Geometric parameters (Å, º)

**Table:**

O1—C12	1.3672 (19)	C11—C12	1.388 (2)
O1—C16	1.427 (2)	C11—H11	0.9500
N1—C2	1.336 (2)	C12—C13	1.394 (2)
N1—C6	1.345 (2)	C13—C14	1.383 (2)
O2—C14	1.362 (2)	C13—H13	0.9500
O2—C17	1.432 (2)	C14—C15	1.398 (2)
C2—N3	1.331 (2)	C15—H15	0.9500
C2—H2	0.9500	C16—H16A	0.9800
N3—C4	1.344 (2)	C16—H16B	0.9800
O3—C18	1.428 (2)	C16—H16C	0.9800
O3—C19	1.438 (2)	C17—H17A	0.9800
C4—C5	1.379 (2)	C17—H17B	0.9800
C4—N9	1.381 (2)	C17—H17C	0.9800
C5—N7	1.392 (2)	C18—C22	1.491 (2)
C5—C6	1.410 (2)	C18—H18	1.0000
N6—C6	1.347 (2)	C19—C20	1.503 (4)
N6—C9	1.453 (2)	C19—H19A	0.9900
N6—H6	0.8800	C19—H19B	0.9900
N7—C8	1.312 (2)	C20—C21	1.504 (3)
C8—N9	1.365 (2)	C20—H20A	0.9900
C8—H8	0.9500	C20—H20B	0.9900
N9—C18	1.451 (2)	C21—C22	1.548 (3)
C9—C10	1.508 (2)	C21—H21A	0.9900
C9—H9A	0.9900	C21—H21B	0.9900
C9—H9B	0.9900	C22—H22A	0.9900
C10—C15	1.381 (2)	C22—H22B	0.9900
C10—C11	1.400 (2)		
			
C12—O1—C16	117.27 (13)	C13—C14—C15	120.74 (16)
C2—N1—C6	118.32 (15)	C10—C15—C14	119.37 (15)
C14—O2—C17	116.38 (13)	C10—C15—H15	120.3
N3—C2—N1	129.90 (16)	C14—C15—H15	120.3
N3—C2—H2	115.0	O1—C16—H16A	109.5
N1—C2—H2	115.0	O1—C16—H16B	109.5
C2—N3—C4	109.91 (15)	H16A—C16—H16B	109.5
C18—O3—C19	111.26 (15)	O1—C16—H16C	109.5
N3—C4—C5	127.40 (15)	H16A—C16—H16C	109.5
N3—C4—N9	126.56 (15)	H16B—C16—H16C	109.5
C5—C4—N9	106.04 (14)	O2—C17—H17A	109.5
C4—C5—N7	110.73 (14)	O2—C17—H17B	109.5
C4—C5—C6	116.57 (15)	H17A—C17—H17B	109.5
N7—C5—C6	132.65 (15)	O2—C17—H17C	109.5
C6—N6—C9	121.98 (14)	H17A—C17—H17C	109.5
C6—N6—H6	119.0	H17B—C17—H17C	109.5
C9—N6—H6	119.0	O3—C18—N9	105.55 (13)
N1—C6—N6	119.01 (15)	O3—C18—C22	112.37 (15)
N1—C6—C5	117.88 (15)	N9—C18—C22	112.58 (15)
N6—C6—C5	123.10 (15)	O3—C18—H18	108.7
C8—N7—C5	103.34 (14)	N9—C18—H18	108.7
N7—C8—N9	114.60 (14)	C22—C18—H18	108.7
N7—C8—H8	122.7	O3—C19—C20	110.3 (2)
N9—C8—H8	122.7	O3—C19—H19A	109.6
C8—N9—C4	105.29 (13)	C20—C19—H19A	109.6
C8—N9—C18	128.47 (14)	O3—C19—H19B	109.6
C4—N9—C18	125.68 (14)	C20—C19—H19B	109.6
N6—C9—C10	113.44 (14)	H19A—C19—H19B	108.1
N6—C9—H9A	108.9	C19—C20—C21	110.10 (18)
C10—C9—H9A	108.9	C19—C20—H20A	109.6
N6—C9—H9B	108.9	C21—C20—H20A	109.6
C10—C9—H9B	108.9	C19—C20—H20B	109.6
H9A—C9—H9B	107.7	C21—C20—H20B	109.6
C15—C10—C11	120.70 (15)	H20A—C20—H20B	108.2
C15—C10—C9	121.21 (15)	C20—C21—C22	109.71 (17)
C11—C10—C9	118.08 (15)	C20—C21—H21A	109.7
C12—C11—C10	119.11 (16)	C22—C21—H21A	109.7
C12—C11—H11	120.4	C20—C21—H21B	109.7
C10—C11—H11	120.4	C22—C21—H21B	109.7
O1—C12—C11	124.41 (15)	H21A—C21—H21B	108.2
O1—C12—C13	114.85 (15)	C18—C22—C21	108.40 (16)
C11—C12—C13	120.74 (15)	C18—C22—H22A	110.0
C14—C13—C12	119.31 (16)	C21—C22—H22A	110.0
C14—C13—H13	120.3	C18—C22—H22B	110.0
C12—C13—H13	120.3	C21—C22—H22B	110.0
O2—C14—C13	124.00 (16)	H22A—C22—H22B	108.4
O2—C14—C15	115.25 (14)		

### Hydrogen-bond geometry (Å, º)

Cg is the centroid of the C10–C15 ring.

**Table:**

*D*—H···*A*	*D*—H	H···*A*	*D*···*A*	*D*—H···*A*
N6—H6···N7^i^	0.88	2.38	3.155 (2)	147
C17—H17*A*···O3^ii^	0.98	2.44	3.216 (3)	136
C8—H8···*Cg*^i^	0.95	2.72	3.506 (2)	142
C21—H21*B*···*Cg*^iii^	0.99	2.93	3.904 (3)	169

Symmetry codes: (i) −*x*+2, −*y*+1, −*z*+1; (ii) *x*, *y*, *z*−1; (iii) −*x*+2, −*y*+2, −*z*+1.
